# Locally Synthetized 17-β-Estradiol Reverses Amyloid-β-42-Induced Hippocampal Long-Term Potentiation Deficits

**DOI:** 10.3390/ijms25031377

**Published:** 2024-01-23

**Authors:** Laura Bellingacci, Jacopo Canonichesi, Miriam Sciaccaluga, Alfredo Megaro, Petra Mazzocchetti, Michela Di Mauro, Cinzia Costa, Massimiliano Di Filippo, Vito Enrico Pettorossi, Alessandro Tozzi

**Affiliations:** Department of Medicine and Surgery, University of Perugia, 06156 Perugia, Italy; laura.bellingacci@unipg.it (L.B.); jacopo.canonichesi@gmail.com (J.C.); miriam.sciaccaluga@unipg.it (M.S.);

**Keywords:** 17β-estradiol, P450-aromatase, 5α-reductase, LTP, synaptic plasticity, estrogen, neurosteroid

## Abstract

Amyloid beta 1-42 (Aβ42) aggregates acutely impair hippocampal long-term potentiation (LTP) of synaptic transmission, and 17β-estradiol is crucial for hippocampal LTP. We tested whether boosting the synthesis of neural-derived 17β-estradiol (nE2) saves hippocampal LTP by the neurotoxic action of Aβ42. Electrophysiological recordings were performed to measure dentate gyrus (DG) LTP in rat hippocampal slices. Using a pharmacological approach, we tested the ability of nE2 to counteract the LTP impairment caused by acute exposure to soluble Aβ42 aggregates. nE2 was found to be required for LTP in DG under physiological conditions. Blockade of steroid 5α-reductase with finasteride, by increasing nE2 synthesis from testosterone (T), completely recovered LTP in slices treated with soluble Aβ42 aggregates. Modulation of the glutamate N-methyl-D aspartate receptor (NMDAR) by memantine effectively rescued the LTP deficit observed in slices exposed to Aβ42, and memantine prevented LTP reduction observed under the blocking of nE2 synthesis. nE2 is able to counteract Aβ42-induced synaptic dysfunction. This effect depends on a rapid, non-genomic mechanism of action of nE2, which may share a common pathway with glutamate NMDAR signaling.

## 1. Introduction

Neurosteroids are a wide range of cholesterol-derived molecules, synthesized de novo within the central nervous system (CNS), able to exert diverse neuronal functions and affect behavior. 17β-Estradiol (E2), one of the most studied estrogenic neuro-active steroids, is known to regulate multiple neuronal molecular signaling systems that are at the basis of synaptic transmission and neural network remodeling and that contribute to learning and memory processing, ultimately affecting cognition [1,2]. Although neuronal- and systemic-derived E2 may contribute simultaneously to synaptic modulation, growing evidence suggests an important contribution of neural E2 (nE2) in the rapid modulation of synaptic transmission and plasticity. Accordingly, nE2 has been demonstrated to exert a pivotal role in the long-term potentiation (LTP) of synaptic transmission in the hippocampus [3,4,5] and in other brain regions [6,7] involved in learning and memory processes [5,8,9,10,11,12]. Dysfunctional E2 signaling has been reported to be dramatically related to several neurological conditions, ranging from psychiatric disorders [13,14] to many neurodegenerative diseases [15,16,17], in which cognitive decline is present to a certain extent. Among others, mild cognitive impairment (MCI), Alzheimer’s disease (AD), or other conditions associated with dementia, display changes in E2 signaling within the CNS [18,19,20] paralleled by changes in cognitive functions. Like most chronic diseases, AD develops slowly from a preclinical phase into a fully expressed clinical syndrome. In AD, large amyloid beta (Aβ) aggregates and plaque deposition are major pathogenic factors of the disease. In this regard, it is known that E2 decreases the generation of Aβ [21] and promotes its degradation, reducing the risk for AD [22]. However, aggregated Aβ accumulation is only a part of a much larger set of pathogenic processes, comprising tau protein hyperphosphorylation or activation of the local immune response, which together initiate cognitive decline. In this pathological setting, soluble Aβ oligomers exist in a dynamic equilibrium with more aggregated fibrillary structures [23,24], and the former seem to be the main toxic species responsible for neural circuit imbalance in critical brain areas such as the hippocampus. Aβ oligomers indeed deeply affect glutamatergic synaptic transmission, altering membrane distribution of glutamate receptors and interfering with intracellular calcium homeostasis, undermining the ability of hippocampal neurons to express LTP, ultimately leading to network dysfunction [25,26,27] and neurological symptoms. Estrogen depletion in mouse models of AD was shown to increase pathological signs and treatment with E2 was shown to exert a protective role against amyloidogenesis and cognitive impairment [28,29,30]. Most of the E2 effects are likely due to the up- or down-regulation of gene transcription, but E2 can also activate rapid intracellular signaling pathways, acting within seconds or minutes through membrane-associated extranuclear receptors [31,32]. E2 rapidly increases synaptic transmission in various brain regions by facilitating glutamate NMDARs activity [33,34,35,36] and inhibiting GABA release [37], thus rapidly modulating the function of neurons [4,38,39,40].

Here, we performed electrophysiological analysis to establish whether nE2 is able to exert rapid modulatory effects on LTP deficits in a rat model of Aβ-induced synaptic dysfunction, resembling synaptic deficits as in an MCI-like condition and AD.

## 2. Materials and Methods

### 2.1. Animals

All procedures involving animals were performed in conformity with the European Directive 2010/63/EU, in accordance with protocols approved by the Animal Care and Use Committee at the University of Perugia, authorization n. 297/2016-PR. All efforts were made to minimize the number of animals used and their suffering. Adult Wistar rats (3-month-old, ~300 g, Charles River, Italy) were used for the experiments; only male animals were used to avoid any possible influence of cyclic estrogenic fluctuation on the induction of synaptic plasticity [41]. Animals were housed at room temperature of about 23 °C with food and water ad libitum and a 12 h light-dark cycle.

### 2.2. Slice Preparation and Electrophysiological Procedures

Rats were decapitated under deep sedation and the brain removed and immersed for 2–3 min in ice-cold artificial cerebrospinal fluid (ACSF) containing (in mM): 126 NaCl, 2.5 KCl, 1.2 MgCl_2_, 1.2 NaH_2_PO_4_, 2.4 CaCl_2_, 10 glucose and 25 NaHCO_3_, continuously bubbled with 95% O_2_ and 5% CO_2_, pH = 7.4. Transversal 400 μm-thick hippocampal slices were obtained using a vibratome (LEICA, VT 1200S) with iced ACSF as the cutting solution. The slices were then transferred to a recovery chamber with oxygenated ACSF at 30 °C for 30 min and then at room temperature (RT) for 1–2 more hours before experimental recordings. Each slice was then transferred into a recording chamber and submerged in ACSF at a constant rate flow of 2.9–3 mL/min at a temperature of 29 °C. Local field excitatory postsynaptic potentials from the hippocampal dentate gyrus (DG) were recorded as previously described [42]. Borosilicate glass microelectrodes filled with 2 mol/L NaCl as recording electrodes (resistance 10–15 MΩ) were placed nearby the granular layer. Electrical responses were evoked by stimulating the perforant pathway at 0.1 Hz (10 μs duration, 20–30 V amplitude) by a pair of bipolar electrodes placed under visual control. An Axoclamp 2B amplifier (Molecular Devices, San Jose, CA, USA) was used for recordings, and traces were filtered at 3 KHz, digitized at 10 KHz and stored in a PC. The postsynaptic responses in the DG included population spikes (PS) that were set at 50% of maximum amplitude. LTP of the PS amplitude was induced by a high-frequency stimulation (HFS) protocol at 100 Hz, consisting of three trains of 1 s, at 5 min intervals.

Drugs were applied by dissolving them to the desired final concentration in oxygenated ACSF and then bath-applied by switching the recording solution to one containing known concentrations of drugs. Total replacement of the medium in the chamber occurred within 1 min. Finasteride (Fin) and E2 were applied at a final concentration of 1 nM, letrozole (Let) at 100 nM and ICI 182,780 (ICI) at 100 nM [7]. For experiments involving memantine (Mem), slices were incubated for 2 h in a chamber containing the drug diluted at 1 μM in oxygenated ACSF [12]. E2, Fin, ICI, Let and Mem were from Tocris Biosciences (Bristol, UK).

### 2.3. Preparation of Aβ1-42 Oligomers and Treatment of Brain Slices

Amyloid β-peptide 1-42 (Aβ42, Innovagen, Lund, Sweden) was initially solubilized with hexafluoro isopropanol (HFIP, Sigma-Aldrich, St. Louis, MO, USA) and incubated at RT for 30 min, resulting in a final peptide concentration of 1 mM. The Aβ42-containing solution was divided in 10 µL aliquots and HFIP was allowed to evaporate overnight in a fume hood. Tubes were then transferred to a SpeedVac and dried down for approximately 1 h. The dried peptide was stored at −80 °C. Immediately before use, aliquots were carefully and completely re-suspended in anhydrous dimethyl sulfoxide (Sigma-Aldrich) by pipette mixing followed by bath sonication for 10 min (5 mM, Aβ42 DMSO stock). Aβ42 oligomers were prepared by diluting 5 mM Aβ42 stock in PBS 0.01 M at pH 7.4, the solution was immediately vortexed for 30 s, then incubated at 4 °C for 24 h [43].

For Aβ42 treatments, some hippocampal slices were moved to an incubation chamber 30 min after cutting, remaining incubated for 2 h in a solution containing oxygenated ACSF enriched with 200 nM fresh Aβ42 oligomers. The slices were then transferred to the recording chamber for electrophysiological recordings [42,44].

### 2.4. Data Analysis and Statistic

Data analysis was performed using Clampfit (Molecular Devices) and GraphPad Prism 8.0.1 (GraphPad software). The time course of the PS amplitude was measured for 15–20 min to obtain a stable, reproducible response to set a baseline and then was measured for subsequent 50 min. Modifications of the PS amplitude induced by drugs or by HFS were expressed as a percentage of the baseline value. The occurrence of LTP was verified by Student’s *t*-test by comparing PS amplitudes 5 min pre-HFS to 40 min post-HFS. Comparisons among different post-HFS PS amplitude time-courses (LTP curves) were evaluated by two-way ANOVA considering the treatment as the main factor. The LTP amplitude corresponded to the PS amplitude measured at 50 min post-HFS. Statistical significance was established at *p* < 0.05. Values given in the text and figures are the mean ± SEM, and n represents the number of slices, 3–4 slices per rat were used for electrophysiological recordings. Aβ42-treated and untreated slices were recorded for each animal.

## 3. Results

### 3.1. Local E2 Synthesis Is Required for the Induction of Long-Term Potentiation in the Dentate Gyrus

**Figure 1:**
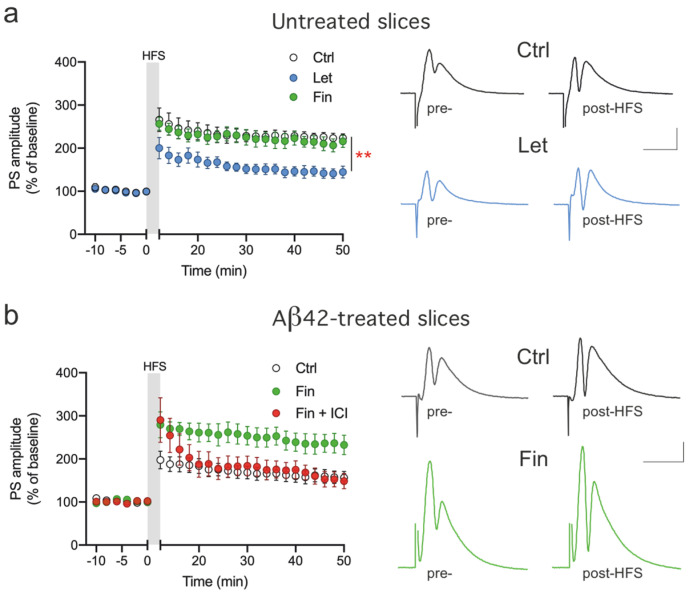
Effect of modulation of nE2 signaling on DG LTP. (**a**) Time-course plot of the mean PS amplitude before and after the HFS protocol to induce LTP in control slices (Ctrl) and in the presence of 100 nM letrozole (Let) or 1 nM finasteride (Fin), (Ctrl, n = 6 vs. Let, n = 5, F_(1,9)_ = 12.95, ** *p* < 0.01; Ctrl vs. Fin, n = 5, *p* > 0.05). Representative pre- and post-HFS traces from a control or a Let-treated slice. (**b**) Time-course plot of the mean PS amplitude before and after the HFS protocol in Aβ42-treated slices after exposure to 1 nM Fin or co-exposure to Fin plus 100 nM ICI 182,780 (ICI) (Aβ42, n = 8 vs. Aβ42 + Fin, n = 5, F_(1,11)_ = 10.2, ** *p* < 0.01; Aβ42 vs. Aβ42 + Fin + ICI, n = 5, *p* > 0.05). Representative pre- and post-HFS traces from Aβ42-treated slices in the absence (Ctrl) or presence of Fin. Scale bars: 1 mV, 10 ms.

To evaluate whether the local synthesis of E2, obtained by the conversion of testosterone (T), could rapidly influence neuronal learning in the DG, we compared the LTP in the presence or absence of the aromatase inhibitor Let. After acquiring a stable PS response, LTP was induced in the control condition or after the bath application of 100 nM Let. We found that in the presence of Let the mean LTP amplitude was reduced by 64% (control, 223.45 ± 9.6%, Let, 144.4 ± 13.8%, *p* < 0.01; Figure 1a), suggesting that the synthesis of E2 is required for the induction of physiological LTP in DG. We also tested the effect of the endogenous production of 5α-dihydrotestosterone (DHT), the major androgenic metabolite of T, on LTP expression in the DG by comparing the LTP in the presence or absence of 1 nM of the steroid 5α-reductase inhibitor Fin. We found that DG LTP did not depend on the presence of endogenous DHT, since LTP measured in the presence of Fin was not significantly different from the control LTP (215.7 ± 14.2%, *p* > 0.05; Figure 1a).

### 3.2. Locally Synthetized E2 Restores LTP in DG of Aβ42-Treated Slices via Direct Interaction with E2 Receptors

**Figure 2:**
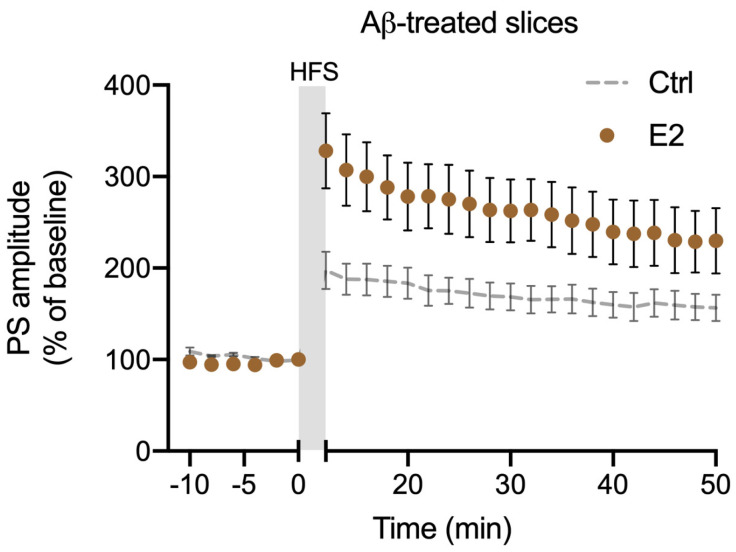
Effect of exogenous 17-β-estradiol (E2) on DG LTP in Aβ42-treated slices. Time-course plot of the mean PS amplitude before and after the HFS protocol to induce LTP in control slices (Ctrl) and in the presence of 1 nM E2 (n = 6).

The Aβ42-induced synaptopathy model we produced by the treatment of rat brain slices with Aβ42 is characterized by an impaired hippocampal LTP [42,44]. To demonstrate the role of locally synthetized E2 in LTP induction in this model, we measured DG LTP after incubation of the slices in ACSF enriched with 200 nM Aβ42 with or without the presence of Fin. We found reduced LTP in slices treated with Aβ42 by 54% with respect to untreated controls (Aβ42, 156.59 ± 14.58%, *p* < 0.01; Figure 1b). Interestingly, in slices exposed to Aβ42 plus 1 nM Fin, LTP impairment was completely prevented (Aβ42 + Fin, 232.57 ± 22.30%, vs. Aβ42 *p* < 0.01; Figure 1b), suggesting that promoting the conversion of T into E2 is sufficient to maintain physiological LTP. To confirm that the restoration of LTP in Aβ42-treated slices in the presence of Fin was in fact due to an increased level of E2, we induced LTP in Aβ-treated slices in the presence of Fin plus 100 nM ICI, a selective antagonist of E2 receptors. In this condition, the mean LTP was reduced by 61%, not significantly different from what was measured in the presence of Aβ42 alone (Aβ42 + Fin + ICI, 148.56 ± 17.81%, vs. Aβ42, *p* > 0.05; Figure 1b), confirming that E2 production is able to prevent LTP impairment in Aβ42-induced synaptopathy. Consistently, exogenous application of 1 nM E2 was able to fully restore DG LTP in Aβ42-treated slices (229.7 ± 35.7%; Figure 2).

### 3.3. nE2-Dependent DG LTP Involves Glutamate NMDA Receptor Function

**Figure 3:**
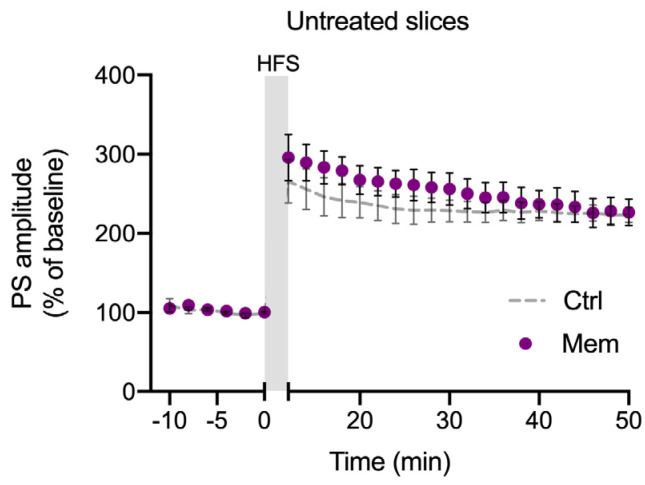
Effect of memantine (Mem) on DG LTP in untreated slices. Time-course plot of the mean PS amplitude before and after the HFS protocol to induce LTP in control slices (Ctrl) and in the presence of 1 μM Mem (n = 6).

**Figure 4:**
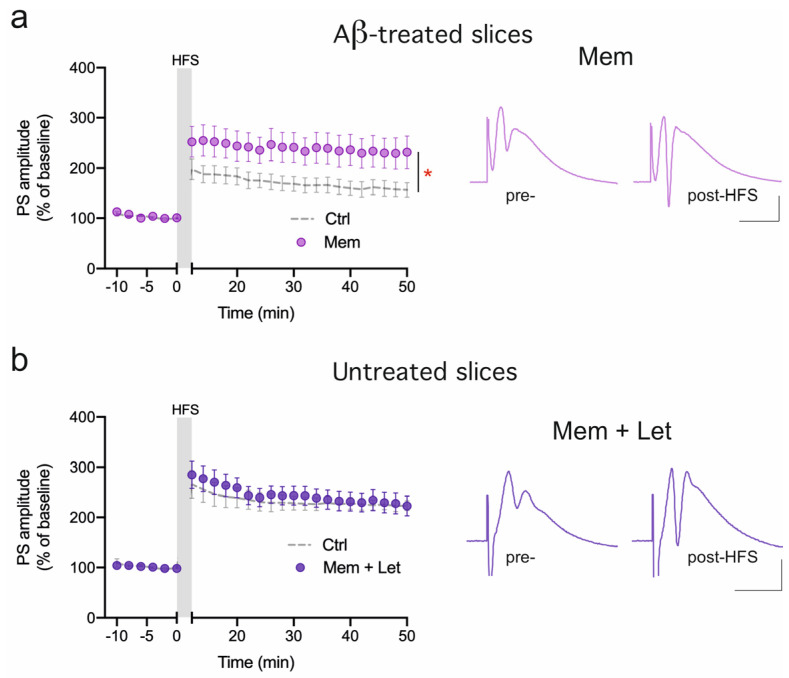
Effect of memantine (Mem) on DG LTP. (**a**) Time-course plot of the mean PS amplitude before and after the HFS protocol in Aβ42 plus 1μM Mem-treated slices (Aβ42 + Mem, 253.56 ± 42.54%, n = 7, vs. Aβ42, dashed time-course (Ctrl), F_(1,13)_ = 4.78, * *p* < 0.05). Representative pre- and post-HFS traces from an Aβ42-treated slice in the presence of Mem. (**b**) Time-course plot of the mean PS amplitude before and after the HFS protocol after the co-exposure of the slices to 100 nM Let plus 1 μM Mem (Mem + Let, 222.80 ± 19.78%, n = 6, vs. Ctrl, dashed time-course, *p* > 0.05). Representative pre- and post-HFS traces from control slices in the presence of Mem plus Let. Scale bars: 1 mV, 10 ms.

We previously found that LTP impairment in DG is fully restored in a genetic model of brain amyloidosis by Mem [12], a noncompetitive NMDAR antagonist that is widely used in clinical practice for AD therapy [45,46]. Here, we tested the effect of Mem on the Aβ42 model of synaptopathy, as it might suggest that the NMDAR plays a role in the modulation of LTP by E2. Thus, LTP was induced in Aβ42-treated slices plus 1μM Mem, a dosage that does not affect physiological DG LTP (226.5 ± 16.84%, Figure 3), showing that this drug is also able to completely prevent the effect of Aβ42 on LTP in acute Aβ42-induced synaptopathy (Aβ42 + Mem, 231.36 ± 32.33%, vs. Aβ42, *p* < 0.05; Figure 4a). To shed light on the possibility that E2 promotes LTP through a mechanism mediated by NMDAR, we induced LTP in hippocampal slices in the presence of 100 nM Let plus 1 μM Mem. In this condition, we found that the exposure to Mem prevented the LTP impairment of DG caused by the blockade of E2 synthesis (Let plus Mem, 222.80 ± 19.78%, *p* > 0.05; Figure 4b) suggesting a convergence of E2 receptor (ER) and NMDAR signaling pathway activation.

## 4. Discussion

**Figure 5:**
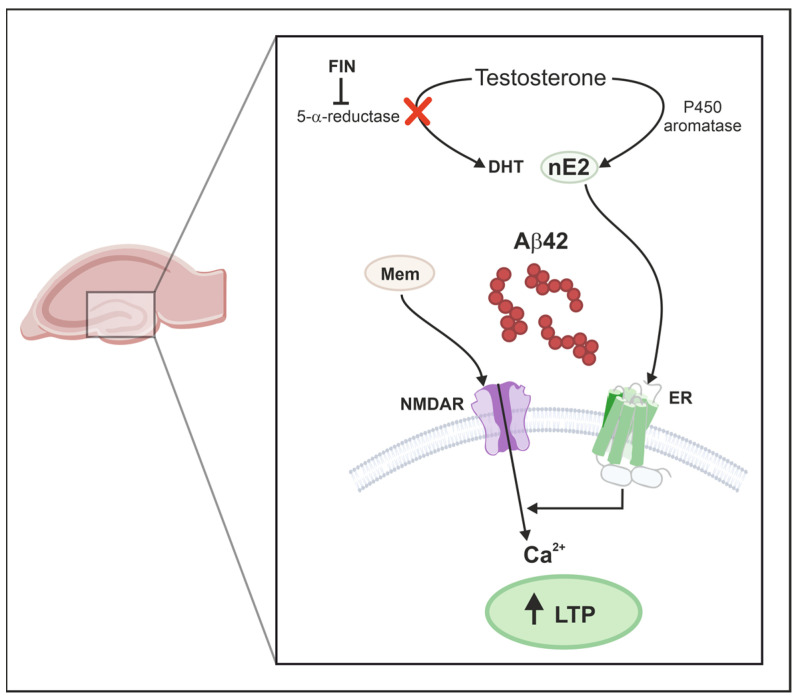
Neural E2 modulation of DG LTP in the model of cerebral amyloidosis. Neural 17-β-estradiol (nE2) levels are increased by finasteride (Fin), an inhibitor of the steroid 5α-reductase (5α-RED) that is responsible for dihydrotestosterone (DHT) production from testosterone (T). T is converted to E2 by P450 aromatase (P450 ARO). In hippocampal slices treated for 2 h with 200 nM of the β-amyloid 1-42 fragment (Aβ42), the high-frequency stimulation protocol is able to induce long-term potentiation (LTP) when the slices of the pathological model are treated with Fin or in the presence of 1 μM of the NMDAR antagonist memantine (Mem). Designed with CorelDRAW Graphics Suite.

E2 of neural origin (nE2) is known to be strongly implicated in LTP induction in different brain regions by exerting rapid, non-genomic effects that are mediated by the activation of its membrane receptors [11,34,39]. The present study, while confirming that nE2 is required for the induction and maintenance of LTP in the hippocampal DG, also demonstrates that it is able to influence LTP expression in a pathological condition such as the synaptic toxicity induced by soluble Aβ42 oligomers. In fact, here we demonstrate for the first time that a pharmacological approach aimed at boosting nE2 levels counteracts Aβ42-induced synaptic dysfunction at the hippocampal DG, rescuing the deficit of the long-term potentiation of synaptic plasticity that characterizes models of cerebral amyloidosis. In the CNS, T can be converted both in E2 by P450 aromatase and in DHT by steroid 5-α-reductase, and it is suggested that the selective inhibition of one enzyme or the other subsequently shifts the local availability of androgen or estrogen [41,47]. Therefore, we inhibited DHT formation with finasteride in order to increase endogenous levels of nE2 in hippocampal slices (Figure 5).

There is evidence that P450 aromatase is expressed at the hippocampal level and that its activation depends on neuronal activity [48,49,50,51]. Performing electrophysiological recordings in rat hippocampal slices, we confirmed that nE2 is required for physiological LTP at the DG, since this form of synaptic plasticity is reduced in the presence of the P450 aromatase inhibitor letrozole, similar to what was observed previously in the CA1 regions by our group [3]. Furthermore, the time window of our recordings (<1 h) strongly suggests that nE2 is able to sustain LTP induction by a rapid non-genomic action [11,52]; indeed, 15–20 min letrozole application appeared sufficient to rapidly reduce the LTP amplitude.

Our experiments demonstrate that the role of nE2 in hippocampal LTP induction is far more relevant when considering pathological conditions. Accordingly, the impairment of hippocampal LTP observed in the presence of Aβ42 oligomers was completely rescued by endogenous nE2 synthesis, obtained by the exposition to finasteride. Of note, finasteride was applied at a nanomolar concentration, a dose that did not alter physiological LTP [41,47]. Interestingly, this low concentration effectively restored DG LTP in Aβ-treated slices without altering the LTP of untreated slices, suggesting increased sensitivity to rapidly synthetized nE2 in the pathological setting of in vitro cerebral amyloidosis induced by Aβ42 aggregates. Accordingly, in this experimental condition, the concurrent E2 receptor blockade abolished the effect obtained with finasteride, while 1 nM E2, a dose comparable with physiological levels of nE2 in the hippocampus of male rats [32,49], was sufficient to restore DG LTP. These findings allow us to hypothesize a possible bidirectional cause-effect relationship between amyloidopathy and altered steroidogenesis in the CNS, also opening the hypothesis that the pharmacological modulation of nE2 levels might have a neuroprotective potential.

Although our study shows that locally synthesized E2 is crucial for the induction and recovery of LTP in the DG, the exact mechanism underlying this phenomenon is not fully understood. The activation of ER by E2 has been reported to induce a rapid increase in spines on granule neurons, significantly enhancing the excitatory input to the DG [52]; accordingly, the loss of hippocampal neuron-derived E2 was shown to significantly decrease the number of dendritic spines and synapses [53]. Our in vitro studies, however, demonstrated that nE2 is able to rescue Aβ42-impaired LTP within minutes, an effect that is far too rapid to be based on granule cells’ spinogenesis. Similar conclusions are in line with findings reporting that LTP impairment preceded hippocampal spine and synapse loss [53,54]. Rapid mechanisms of action in which nE2 can play a role in LTP include its influence on glutamatergic and GABAergic transmission [37,52,55,56,57]. Since hippocampal LTP is mediated by the activation of NMDARs [58], nE2 likely facilitates LTP by interacting with these receptors, as reported for exogenous E2 [59,60,61]. Moreover, nE2-dependent LTP induction has been shown to rely on the interaction between ERs and NMDARs signaling cascades in different hippocampal regions [35,59,60,62]. Thus, we hypothesized in hippocampal DG a similar convergent interaction between nE2 signaling and activation of the NMDAR intracellular pathway (Figure 5). Indeed, our experiments demonstrate that in control conditions, the LTP induced in the presence of letrozole and memantine is not altered, suggesting that NMDARs’ modulation acts downstream of ERs. 

Our results are in line with the observations of Tanaka and colleagues showing that endogenously synthesized E2 constitutively enhances NMDAR function through synaptic ER [63,64]. Moreover, numerous studies have demonstrated that Aβ oligomers directly alter the function of NMDARs. In particular, Aβ oligomers specifically activated the extra-synaptic NMDARs subpopulation, responsible for glutamate excitotoxicity and cell death [65,66], and reduced the synaptic sub-population of NMDARs [67,68,69], disrupting the balance between synaptic and extra-synaptic NMDARs [70]. The non-competitive NMDAR antagonist memantine, widely used in moderate and severe dementia, antagonized Aβ-induced negative effects [71] by acting on extra-synaptic NMDARs [72,73,74]. In this scenario, we can hypothesize that locally synthesized E2 can recover hippocampal LTP by acting on the balance between synaptic and extra-synaptic NMDARs’ functionality. 

Although further investigation, including measurements of single neurons, may help to illustrate the specific interaction between nE2 and NMDAR in DG LTP, we can conclude that the gatekeeping function of the DG to filter incoming activity in the hippocampus can be modulated by nE2 under both physiological and pathological conditions through the modulation of NMDARs. Our results confirm the pivotal role of locally synthesized E2 in mediating long-term changes in synaptic strength, highlighting the importance of its effect on memory and learning mechanisms. Moreover, our data highlight the importance of nE2 as a possible neuroprotective agent in cerebral amyloidosis, able to counteract the early loss of synaptic plasticity associated with Aβ aggregates’ accumulation. 

## Data Availability

The data presented in this study are available on request from the corresponding author.

## References

[1] Brann D.W., Lu Y., Wang J., Zhang Q., Thakkar R., Sareddy G.R., Pratap U.P., Tekmal R.R., Vadlamudi R.K. (2022). Brain-derived estrogen and neural function. Neurosci. Biobehav. Rev..

[2] Marbouti L., Zahmatkesh M., Riahi E., Sadr S.S. (2020). Inhibition of brain 17β-estradiol synthesis by letrozole induces cognitive decline in male and female rats. Neurobiol. Learn. Mem..

[3] Grassi S., Tozzi A., Costa C., Tantucci M., Colcelli E., Scarduzio M., Calabresi P., Pettorossi V.E. (2011). Neural 17β-estradiol facilitates long-term potentiation in the hippocampal CA1 region. Neuroscience.

[4] Hasegawa Y., Hojo Y., Kojima H., Ikeda M., Hotta K., Sato R., Ooishi Y., Yoshiya M., Chung B.C., Yamazaki T. (2015). Estradiol rapidly modulates synaptic plasticity of hippocampal neurons: Involvement of kinase networks. Brain Res..

[5] Tozzi A., Durante V., Manca P., Di Mauro M., Blasi J., Grassi S., Calabresi P., Kawato S., Pettorossi V.E. (2019). Bidirectional Synaptic Plasticity Is Driven by Sex Neurosteroids Targeting Estrogen and Androgen Receptors in Hippocampal CA1 Pyramidal Neurons. Front. Cell. Neurosci..

[6] Bender R.A., Zhou L., Vierk R., Brandt N., Keller A., Gee C.E., Schäfer M.K., Rune G.M. (2017). Sex-Dependent Regulation of Aromatase-Mediated Synaptic Plasticity in the Basolateral Amygdala. J. Neurosci..

[7] Tozzi A., de Iure A., Tantucci M., Durante V., Quiroga-Varela A., Giampà C., Di Mauro M., Mazzocchetti P., Costa C., Di Filippo M. (2015). Endogenous 17β-estradiol is required for activity-dependent long-term potentiation in the striatum: Interaction with the dopaminergic system. Front. Cell. Neurosci..

[8] Dieni C.V., Contemori S., Biscarini A., Panichi R. (2020). De Novo Synthesized Estradiol: A Role in Modulating the Cerebellar Function. Int. J. Mol. Sci..

[9] Diotel N., Charlier T.D., Lefebvre d’Hellencourt C., Couret D., Trudeau V.L., Nicolau J.C., Meilhac O., Kah O., Pellegrini E. (2018). Steroid Transport, Local Synthesis, and Signaling within the Brain: Roles in Neurogenesis, Neuroprotection, and Sexual Behaviors. Front. Neurosci..

[10] Luine V., Frankfurt M. (2020). Estrogenic regulation of memory: The first 50 years. Horm. Behav..

[11] Tozzi A., Bellingacci L., Pettorossi V.E. (2020). Rapid Estrogenic and Androgenic Neurosteroids Effects in the Induction of Long-Term Synaptic Changes: Implication for Early Memory Formation. Front. Neurosci..

[12] Tozzi A., Sclip A., Tantucci M., de Iure A., Ghiglieri V., Costa C., Di Filippo M., Borsello T., Calabresi P. (2015). Region- and age-dependent reductions of hippocampal long-term potentiation and NMDA to AMPA ratio in a genetic model of Alzheimer’s disease. Neurobiol. Aging.

[13] Bangasser D.A., Valentino R.J. (2014). Sex differences in stress-related psychiatric disorders: Neurobiological perspectives. Front. Neuroendocrinol..

[14] Hodes G.E., Epperson C.N. (2019). Sex Differences in Vulnerability and Resilience to Stress Across the Life Span. Biol. Psychiatry.

[15] Jett S., Malviya N., Schelbaum E., Jang G., Jahan E., Clancy K., Hristov H., Pahlajani S., Niotis K., Loeb-Zeitlin S. (2022). Endogenous and Exogenous Estrogen Exposures: How Women’s Reproductive Health Can Drive Brain Aging and Inform Alzheimer’s Prevention. Front. Aging Neurosci..

[16] Nilsen J. (2008). Estradiol and neurodegenerative oxidative stress. Front. Neuroendocrinol..

[17] Sahab-Negah S., Hajali V., Moradi H.R., Gorji A. (2020). The Impact of Estradiol on Neurogenesis and Cognitive Functions in Alzheimer’s Disease. Cell. Mol. Neurobiol..

[18] Jett S., Schelbaum E., Jang G., Boneu Yepez C., Dyke J.P., Pahlajani S., Diaz Brinton R., Mosconi L. (2022). Ovarian steroid hormones: A long overlooked but critical contributor to brain aging and Alzheimer’s disease. Front. Aging Neurosci..

[19] Oveisgharan S., Yang J., Yu L., Burba D., Bang W., Tasaki S., Grodstein F., Wang Y., Zhao J., De Jager P.L. (2023). Estrogen Receptor Genes, Cognitive Decline, and Alzheimer Disease. Neurology.

[20] Yue X., Lu M., Lancaster T., Cao P., Honda S., Staufenbiel M., Harada N., Zhong Z., Shen Y., Li R. (2005). Brain estrogen deficiency accelerates Abeta plaque formation in an Alzheimer’s disease animal model. Proc. Natl. Acad. Sci. USA.

[21] Xu H., Wang R., Zhang Y.W., Zhang X. (2006). Estrogen, beta-amyloid metabolism/trafficking, and Alzheimer’s disease. Ann. N. Y. Acad. Sci..

[22] Liang K., Yang L., Yin C., Xiao Z., Zhang J., Liu Y., Huang J. (2010). Estrogen stimulates degradation of beta-amyloid peptide by up-regulating neprilysin. J. Biol. Chem..

[23] Hong S., Ostaszewski B.L., Yang T., O’Malley T.T., Jin M., Yanagisawa K., Li S., Bartels T., Selkoe D.J. (2014). Soluble Abeta oligomers are rapidly sequestered from brain ISF in vivo and bind GM1 ganglioside on cellular membranes. Neuron.

[24] Shankar G.M., Li S., Mehta T.H., Garcia-Munoz A., Shepardson N.E., Smith I., Brett F.M., Farrell M.A., Rowan M.J., Lemere C.A. (2008). Amyloid-beta protein dimers isolated directly from Alzheimer’s brains impair synaptic plasticity and memory. Nat. Med..

[25] Li S., Selkoe D.J. (2020). A mechanistic hypothesis for the impairment of synaptic plasticity by soluble Aβ oligomers from Alzheimer’s brain. J. Neurochem..

[26] Reiss A.B., Arain H.A., Stecker M.M., Siegart N.M., Kasselman L.J. (2018). Amyloid toxicity in Alzheimer’s disease. Rev. Neurosci..

[27] Sciaccaluga M., Megaro A., Bellomo G., Ruffolo G., Romoli M., Palma E., Costa C. (2021). An Unbalanced Synaptic Transmission: Cause or Consequence of the Amyloid Oligomers Neurotoxicity?. Int. J. Mol. Sci..

[28] Vegeto E., Belcredito S., Ghisletti S., Meda C., Etteri S., Maggi A. (2006). The endogenous estrogen status regulates microglia reactivity in animal models of neuroinflammation. Endocrinology.

[29] Yun J., Yeo I.J., Hwang C.J., Choi D.Y., Im H.S., Kim J.Y., Choi W.R., Jung M.H., Han S.B., Hong J.T. (2018). Estrogen deficiency exacerbates Aβ-induced memory impairment through enhancement of neuroinflammation, amyloidogenesis and NF-ĸB activation in ovariectomized mice. Brain Behav. Immun..

[30] Zhu Y., Zhang Q., Zhang W., Li N., Dai Y., Tu J., Yang F., Brann D.W., Wang R. (2017). Protective Effect of 17beta-Estradiol Upon Hippocampal Spine Density and Cognitive Function in an Animal Model of Vascular Dementia. Sci. Rep..

[31] Kawato S. (2004). Endocrine disrupters as disrupters of brain function: A neurosteroid viewpoint. Environ. Sci..

[32] Mukai H., Takata N., Ishii H.T., Tanabe N., Hojo Y., Furukawa A., Kimoto T., Kawato S. (2006). Hippocampal synthesis of estrogens and androgens which are paracrine modulators of synaptic plasticity: Synaptocrinology. Neuroscience.

[33] Dominguez R., Liu R., Baudry M. (2007). 17-Beta-estradiol-mediated activation of extracellular-signal regulated kinase, phosphatidylinositol 3-kinase/protein kinase B-Akt and N-methyl-D-aspartate receptor phosphorylation in cortical synaptoneurosomes. J. Neurochem..

[34] Grassi S., Frondaroli A., Scarduzio M., Dutia M.B., Dieni C., Pettorossi V.E. (2010). Effects of 17beta-estradiol on glutamate synaptic transmission and neuronal excitability in the rat medial vestibular nuclei. Neuroscience.

[35] Smith C.C., Smith L.A., Bredemann T.M., McMahon L.L. (2016). 17β estradiol recruits GluN2B-containing NMDARs and ERK during induction of long-term potentiation at temporoammonic-CA1 synapses. Hippocampus.

[36] Wong M., Moss R.L. (1992). Long-term and short-term electrophysiological effects of estrogen on the synaptic properties of hippocampal CA1 neurons. J. Neurosci..

[37] Rudick C.N., Woolley C.S. (2001). Estrogen regulates functional inhibition of hippocampal CA1 pyramidal cells in the adult female rat. J. Neurosci..

[38] Evinger A.J., Levin E.R. (2005). Requirements for estrogen receptor alpha membrane localization and function. Steroids.

[39] Hojo Y., Kawato S. (2018). Neurosteroids in Adult Hippocampus of Male and Female Rodents: Biosynthesis and Actions of Sex Steroids. Front. Endocrinol..

[40] Murakami G., Hojo Y., Kato A., Komatsuzaki Y., Horie S., Soma M., Kim J., Kawato S. (2018). Rapid nongenomic modulation by neurosteroids of dendritic spines in the hippocampus: Androgen, oestrogen and corticosteroid. J. Neuroendocrinol..

[41] Di Mauro M., Tozzi A., Calabresi P., Pettorossi V.E., Grassi S. (2017). Different synaptic stimulation patterns influence the local androgenic and estrogenic neurosteroid availability triggering hippocampal synaptic plasticity in the male rat. Eur. J. Neurosci..

[42] Bellingacci L., Tallarico M., Mancini A., Megaro A., De Caro C., Citraro R., De Sarro G., Tozzi A., Di Filippo M., Sciaccaluga M. (2023). Non-competitive AMPA glutamate receptors antagonism by perampanel as a strategy to counteract hippocampal hyper-excitability and cognitive deficits in cerebral amyloidosis. Neuropharmacology.

[43] Stine W.B., Dahlgren K.N., Krafft G.A., LaDu M.J. (2003). In vitro characterization of conditions for amyloid-beta peptide oligomerization and fibrillogenesis. J. Biol. Chem..

[44] Costa C., Parnetti L., D’Amelio M., Tozzi A., Tantucci M., Romigi A., Siliquini S., Cavallucci V., Di Filippo M., Mazzocchetti P. (2016). Epilepsy, amyloid-β, and D1 dopamine receptors: A possible pathogenetic link?. Neurobiol. Aging.

[45] Danysz W., Parsons C.G. (2003). The NMDA receptor antagonist memantine as a symptomatological and neuroprotective treatment for Alzheimer’s disease: Preclinical evidence. Int. J. Geriatr. Psychiatry.

[46] Reisberg B., Doody R., Stöffler A., Schmitt F., Ferris S., Möbius H.J. (2003). Memantine in moderate-to-severe Alzheimer’s disease. N. Engl. J. Med..

[47] Di Mauro M., Tozzi A., Calabresi P., Pettorossi V.E., Grassi S. (2015). Neo-synthesis of estrogenic or androgenic neurosteroids determine whether long-term potentiation or depression is induced in hippocampus of male rat. Front. Cell. Neurosci..

[48] Balthazart J., Cornil C.A., Taziaux M., Charlier T.D., Baillien M., Ball G.F. (2006). Rapid changes in production and behavioral action of estrogens. Neuroscience.

[49] Hojo Y., Hattori T.A., Enami T., Furukawa A., Suzuki K., Ishii H.T., Mukai H., Morrison J.H., Janssen W.G., Kominami S. (2004). Adult male rat hippocampus synthesizes estradiol from pregnenolone by cytochromes P45017alpha and P450 aromatase localized in neurons. Proc. Natl. Acad. Sci. USA.

[50] Hojo Y., Murakami G., Mukai H., Higo S., Hatanaka Y., Ogiue-Ikeda M., Ishii H., Kimoto T., Kawato S. (2008). Estrogen synthesis in the brain—Role in synaptic plasticity and memory. Mol. Cell. Endocrinol..

[51] Kimoto T., Tsurugizawa T., Ohta Y., Makino J., Tamura H., Hojo Y., Takata N., Kawato S. (2001). Neurosteroid synthesis by cytochrome p450-containing systems localized in the rat brain hippocampal neurons: N-methyl-D-aspartate and calcium-dependent synthesis. Endocrinology.

[52] Hojo Y., Munetomo A., Mukai H., Ikeda M., Sato R., Hatanaka Y., Murakami G., Komatsuzaki Y., Kimoto T., Kawato S. (2015). Estradiol rapidly modulates spinogenesis in hippocampal dentate gyrus: Involvement of kinase networks. Horm. Behav..

[53] Lu Y., Sareddy G.R., Wang J., Wang R., Li Y., Dong Y., Zhang Q., Liu J., O’Connor J.C., Xu J. (2019). Neuron-Derived Estrogen Regulates Synaptic Plasticity and Memory. J. Neurosci..

[54] Vierk R., Glassmeier G., Zhou L., Brandt N., Fester L., Dudzinski D., Wilkars W., Bender R.A., Lewerenz M., Gloger S. (2012). Aromatase inhibition abolishes LTP generation in female but not in male mice. J. Neurosci..

[55] Mukai H., Tsurugizawa T., Murakami G., Kominami S., Ishii H., Ogiue-Ikeda M., Takata N., Tanabe N., Furukawa A., Hojo Y. (2007). Rapid modulation of long-term depression and spinogenesis via synaptic estrogen receptors in hippocampal principal neurons. J. Neurochem..

[56] Murphy D.D., Cole N.B., Greenberger V., Segal M. (1998). Estradiol increases dendritic spine density by reducing GABA neurotransmission in hippocampal neurons. J. Neurosci..

[57] Wojtowicz T., Lebida K., Mozrzymas J.W. (2008). 17beta-estradiol affects GABAergic transmission in developing hippocampus. Brain Res..

[58] Bliss T.V., Collingridge G.L. (1993). A synaptic model of memory: Long-term potentiation in the hippocampus. Nature.

[59] Foy M.R. (2001). 17beta-estradiol: Effect on CA1 hippocampal synaptic plasticity. Neurobiol. Learn. Mem..

[60] Smith C.C., McMahon L.L. (2006). Estradiol-induced increase in the magnitude of long-term potentiation is prevented by blocking NR2B-containing receptors. J. Neurosci..

[61] Oberlander J.G., Woolley C.S. (2016). 17beta-Estradiol Acutely Potentiates Glutamatergic Synaptic Transmission in the Hippocampus through Distinct Mechanisms in Males and Females. J. Neurosci..

[62] Clements L., Harvey J. (2020). Activation of oestrogen receptor alpha induces a novel form of LTP at hippocampal temporoammonic-CA1 synapses. Br. J. Pharmacol..

[63] Tanaka M., Sokabe M. (2012). Continuous de novo synthesis of neurosteroids is required for normal synaptic transmission and plasticity in the dentate gyrus of the rat hippocampus. Neuropharmacology.

[64] Tanaka M., Sokabe M. (2013). Bidirectional modulatory effect of 17β-estradiol on NMDA receptors via ERα and ERβ in the dentate gyrus of juvenile male rats. Neuropharmacology.

[65] Léveillé F., El Gaamouch F., Gouix E., Lecocq M., Lobner D., Nicole O., Buisson A. (2008). Neuronal viability is controlled by a functional relation between synaptic and extrasynaptic NMDA receptors. FASEB J..

[66] Stanika R.I., Pivovarova N.B., Brantner C.A., Watts C.A., Winters C.A., Andrews S.B. (2009). Coupling diverse routes of calcium entry to mitochondrial dysfunction and glutamate excitotoxicity. Proc. Natl. Acad. Sci. USA.

[67] Geddes J.W., Chang-Chui H., Cooper S.M., Lott I.T., Cotman C.W. (1986). Density and distribution of NMDA receptors in the human hippocampus in Alzheimer’s disease. Brain Res..

[68] Hynd M.R., Scott H.L., Dodd P.R. (2001). Glutamate(NMDA) receptor NR1 subunit mRNA expression in Alzheimer’s disease. J. Neurochem..

[69] Procter A.W., Stirling J.M., Stratmann G.C., Cross A.J., Bowen D.M. (1989). Loss of glycine-dependent radioligand binding to the N-methyl-D-aspartate-phencyclidine receptor complex in patients with Alzheimer’s disease. Neurosci. Lett..

[70] Wang M., Yang Y., Wang C.J., Gamo N.J., Jin L.E., Mazer J.A., Morrison J.H., Wang X.J., Arnsten A.F. (2013). NMDA receptors subserve persistent neuronal firing during working memory in dorsolateral prefrontal cortex. Neuron.

[71] Talantova M., Sanz-Blasco S., Zhang X., Xia P., Akhtar M.W., Okamoto S., Dziewczapolski G., Nakamura T., Cao G., Pratt A.E. (2013). Aβ induces astrocytic glutamate release, extrasynaptic NMDA receptor activation, and synaptic loss. Proc. Natl. Acad. Sci. USA.

[72] Wang R., Reddy P.H. (2017). Role of Glutamate and NMDA Receptors in Alzheimer’s Disease. J. Alzheimers Dis..

[73] Wu Y.N., Johnson S.W. (2015). Memantine selectively blocks extrasynaptic NMDA receptors in rat substantia nigra dopamine neurons. Brain Res..

[74] Xia P., Chen H.S., Zhang D., Lipton S.A. (2010). Memantine preferentially blocks extrasynaptic over synaptic NMDA receptor currents in hippocampal autapses. J. Neurosci..

